# Prevalence and Serological Diagnosis of Relapse in Paracoccidioidomycosis Patients

**DOI:** 10.1371/journal.pntd.0002834

**Published:** 2014-05-01

**Authors:** Tatiane Fernanda Sylvestre, Luciane Regina Franciscone Silva, Ricardo de Souza Cavalcante, Daniela Vanessa Moris, James Venturini, Adriana Pardini Vicentini, Lídia Raquel de Carvalho, Rinaldo Poncio Mendes

**Affiliations:** 1 Tropical Diseases Department - Faculdade de Medicina de Botucatu – Universidade Estadual Paulista (UNESP), Botucatu, São Paulo, Brazil; 2 Mycoses Immunodiagnostic Laboratory, Immunology Section, Adolfo Lutz Institute, São Paulo, Brazil; 3 Universidade do Oeste Paulista – UNOESTE, Presidente Prudente, São Paulo, Brazil; 4 Laboratory of Experimental Immunology, Department of Biological Science, Faculty of Science, São Paulo State University – UNESP, São Paulo, Brazil; 5 Instituto de Biociências de Botucatu – UNESP, Botucatu, São Paulo, Brazil; University of California San Diego School of Medicine, United States of America

## Abstract

A review of 400 clinical records of paracoccidioidomycosis (PCM) patients, 93 with the acute/subacute (AF) and 307 with the chronic form (CF), attended from 1977 to 2011, selected as to the schedule of release for study by the Office of Medical Records at the University Hospital of the Faculdade de Medicina de Botucatu – São Paulo State University – UNESP, was performed to detect cases in relapse. The control of cure was performed by clinical and serological evaluation using the double agar gel immunodiffusion test (DID). In the diagnosis of relapse, DID, enzyme-linked immunosorbent assay (ELISA) and immunoblotting assay (IBgp70 and IBgp43) were evaluated. Out of 400 patients, 21 (5.2%) went through relapse, 18 of them were male and 3 were female, 6∶1 male/female ratio. Out of the 21 patients in relapse, 15 (4.8%) showed the CF, and 6 (6.4%) the AF (p>0.05). The sensitivity of DID and ELISA before treatment was the same (76.1%). DID presented higher sensitivity in pre-treatment (80%) than at relapse (45%; p = 0.017), while ELISA showed the same sensitivity (80% *vs* 65%; p = 0.125). The serological methods for identifying PCM patients in relapse showed low rates of sensitivity, from 12.5% in IBgp70 to 65.0% in IBgp43 identification and 68.8% in ELISA. The sensitivity of ELISA in diagnosing PCM relapse showed a strong tendency to be higher than DID (p = 0.06) and is equal to IBgp43 (p = 0.11). In sum, prevalence of relapse was not high in PCM patients whose treatment duration was based on immunological parameters. However, the used methods for serological diagnosis present low sensitivity. While more accurate serological methods are not available, we pay special attention to the mycological and histopathological diagnosis of PCM relapse. Hence, direct mycological, cytopathological, and histopathological examinations and isolation in culture for *P. brasiliensis* must be appropriately and routinely performed when the hypothesis of relapse is considered.

## Author Summary

Paracoccidioidomycosis (PCM) is a systemic mycosis caused by fungi of the *Paracoccidioides brasiliensis* and *Paracoccidioides lutzii* complexes, which live in the soil and affect mainly rural workers in the most productive period of their lives. PCM can relapse after effective treatment because quiescent fungi can reactivate. However, the physician has to differentiate a relapse from another disease. In the diagnosis of relapse, the classical double agar gel immunodiffusion test (DID) and the modern enzyme-linked immunosorbent assay (ELISA) and immunoblotting (IB) were evaluated. The frequency of PCM relapse and its diagnosis were the objective of this study. The prevalence of relapse was low and the new serological tests presented a little higher sensitivity than DID. Since the serological tests presented only a moderate sensitivity, direct mycological, cytopathological, and histopathological examinations and isolation in culture for the fungi must be appropriately and routinely performed when the hypothesis of relapse is considered.

## Introduction

Paracoccidioidomycosis (PCM) is a systemic mycosis caused by thermo-dimorphic fungi from the *Paracoccidioides brasiliensis* complex and the *Paracoccidioides lutzii* complex [1]. Confined to Latin America, PCM is endemic to the area extending from Mexico to Argentina [2]. Although incomplete, the available data indicate a higher incidence of such mycosis in Brazil, where they are frequently diagnosed in the State of São Paulo [3].

PCM is known to be able to reactivate despite effective treatment because of quiescent fungi remain and disease relapse is possible. However, a few studies have investigated the relapse of paracoccidioidomycosis. A study that was conducted with 58 patients who were infected with paracoccidioidomycosis and treated with itraconazole indicated that there was a relapse in 8 (13.8%) of the cases, where 50.0% of them occurred after 36 months of discontinued treatment [4].

The gold standard for diagnosing PCM is either the direct visualization of characteristic multiple-budding cells in biological fluids and tissue sections, or fungus isolation from clinical specimens [5]. Serological tests are useful for diagnosis, severity assessment and follow-up, especially the double agar gel immunodiffusion test (DID) [6].

The ELISA (Enzyme-Linked Immunosorbent Assay) test has been the subject of a number of publications in regard to the detection of circulating antibodies PCM patients [7], although it has not been included in most clinical laboratories.

This paper is aimed at evaluating the ELISA test and its ability to detect antibodies against *Paracoccidioides brasiliensis* in PCM patients in relapse.

## Materials and Methods

A prospective study was conducted with patients who were receiving medical attention at the Paracoccidioidomycosis Outpatient Service in the Infectious Diseases Center of the University Hospital in Botucatu Medical School – UNESP. The need to adhere to the treatment given, as well as, when indicated, the suppression of alcohol intake and smoking, was reiterated in all of the outpatient visits.

### Ethics statement

This study was approved by the Research Ethics Committee of FMB-UNESP. Written informed consent was obtained from all participants. In this study IRB was signed by all the adult patients and by one of the parents of the children. We had no IRB signed by the closest relative or the legal representative.

### Population under study

The criteria for the inclusion of the patients in the study were as follows: (A) Upon diagnosis: (a) PCM confirmed cases, characterized by the presence of a suggestive clinical condition and the identification of typical forms of *P. brasiliensis* yeast phase in one or more clinical materials; (b) PCM probable cases, characterized by the presence of suggestive clinical conditions and specific serum antibodies detected by the results of the DID test. (B) After treatment: patients showing PCM relapse, characterized by the recurrence of signs and symptoms indicative of PCM, with or without the identification of the typical forms of *P. brasiliensis* yeast phase in any clinical specimen, and/or the serology reaction to the DID test. Those in this group have also been given the appropriate therapy and have shown clinical cure, experienced a normalization of their erythrocyte sedimentation rate (ESR) and a serology regression to negative values, and have continued antifungal treatment for at least one year after serological cure.

The exclusion criteria were as follows: presence of other systemic diseases of infectious, inflammatory or neoplastic source, such as co-morbidity; pregnancy; lactation; history of hypersensitivity or severe side effects in response to azole or cotrimoxazole; concomitant use of medicines that can interact with such antifungals or change their serum levels or concomitant use of other antifungals.

Eight patients with mycologically confirmed and PCM who did not relapse constituted the control group; 122 serum samples from these patients were serologically evaluated by the double agar gel immunodiffusion test (DID) and the enzyme-linked immunosorbent assay (ELISA).

### Classification of clinical forms

The categorization of patients and the assessment of disease severity in each patient were carried out according to Mendes [8] and the Paracoccidioidomycosis Surveillance and Control Guideline [6] by the infectious disease MD responsible for attending the patients.

### Review of medical records

A review of 400 clinical records of PCM patients, 93 of whom with the acute/subacute (AF) and 307 with the chronic form (CF), attended from 1977 to 2011 selected as to the order of release for study by the Office of Medical Records in the University Hospital of the Faculdade de Medicina de Botucatu – São Paulo State University – UNESP, was performed to detected cases patients in relapse. A standard form was filled in per patient containing their name, sex, age, enrollment code at the Hospital, date of admission to the service, presence of previous treatment, clinical form, treatment start date, and results of diagnostic and serological tests.

Likewise, a review of the serological records of PCM patients was conducted by the Tropical Diseases & Mycology Research Laboratory – Department of Infectious and Parasitic Diseases, in Botucatu Medical School – UNESP.

### Appropriate treatment and definition of relapse cases

Treatment was deemed appropriate if the signs and symptoms of the disease were gone, there was a normalization of the erythrocyte sedimentation rate (ESR) and a negative reaction to DID could be observed during one year of antifungal medicine administration and for at least one year without such therapy.

The categorization of cases of relapse was characterized by the reappearance of signs and symptoms that were compatible with PCM, with or without the identification of the typical *P. brasiliensis* yeast forms in any clinical specimen, or the reaction to DID after the appropriate treatment in patients with confirmed or probable PCM. Five types of relapse have been taken into account: (a) clinical, serological and mycological, characterized by the reappearance of signs and symptoms compatible with PCM, with or without identification of the typical *P. brasiliensis* yeast forms in any clinical specimen, and a positive DID serology test; (b) clinical and serological, characterized by the reappearance of signs and symptoms compatible with PCM and a positive DID serology test; (c) clinical and mycological, characterized by the reappearance of signs and symptoms compatible with PCM, with the identification of the typical *P. brasiliensis* yeast s in their common forms in any clinical specimen; (d) clinical, characterized by the reappearance of the clinical signs of PCM, responsiveness to sulfamethoxazole-trimethoprim combination (TMP-SMX); and (e) serological, characterized only by a positive DID response without clinical signs.

### Antigen used

The *P. brasiliensis* 113 (Pb-113) yeast phase culture filtrate, prepared at the Clinical Mycology Laboratory of Araraquara Pharmaceutical Sciences College – UNESP, was used to run DID, ELISA and *immunoblotting* (IB) tests.

### Double agar gel immunodiffusion test

The serum levels of anti-Pb antibodies were determined through a DID test which was run according to the specifications of Restrepo [9] at the Tropical Diseases Experimental Laboratory in Botucatu Medical School – UNESP.

Non-diluted sera were tested first and then diluted by ½, with serial dilutions (×2).

For each test, positive and negative control sera were included.

### ELISA (enzyme-linked immunosorbent assay) – indirect method

The serum levels of anti-Pb antibodies were determined through an ELISA test [10]–[11], which was run at the Tropical Diseases Experimental Laboratory in Botucatu Medical School – UNESP, according to the standard protocol set forth by the Mycosis Immunodiagnosis Laboratory of Adolfo Lutz Institute (São Paulo, Brazil).

The cutoff point was determined by drawing the ROC (receiver operator characteristic) curve for 200 PCM patients and 100 healthy subjects (control subjects) from Botucatu Blood Bank. The final end point was statistically defined according to the specifications made by Frey et al. [12] for a 95% confidence interval, and was equal to 0.710 optical density.

### Immunoblotting assay evaluation

Such serological techniques were conducted at the Mycosis Immunodiagnosis Laboratory of Adolfo Lutz Institute, in São Paulo, Brazil.

Electrophoresis that involved the transfer of proteins contained in polyacrylamide gels to nitrocellulose membranes was carried out according to a methodology described by Towbin et al. [13], as amended by Passos [14].

### Statistical analysis of results

A 2×2 comparison of the sensitivity of the serological methods that were used was carried out through a binomial test, according to the specifications made by Siegel [15].

A comparison of the curves representing the time to achieve serological response in different groups of patients and serological techniques was conducted by using the ranks for the two methods, and regression was calculated for each of them, which were then compared to both regressions, through F-test [16].

A kappa coefficient was ascertained by means of SAS – statistical analysis system, Version 6.12, SAS Institute Inc., USA. The kappa coefficient was interpreted as follows: (a) mild, when lower than 0.4; (b) moderate, from 0.4 to 0.79; (c) strong, from 0.8 to 0.99; and (d) perfect, when equal to 1.0.

For each such statistic test, differences were set up at ***p***≤0.05.

## Results

### PCM relapse patients characterization

Out of 400 patients, 21 (5.2%) went through relapse, 3 of which (14.3%) were female and 18 (85.7%) were male, resulting in a male/female ratio of 6∶1. The prevalence of male patients was 85.7% among the patients in relapse, 88.1% among the patients who did not relapse and 88.0% among the 400 studied patients, with no difference between patients in relapse or not (p = 0.72).

Out of the 21 patients who experienced a relapse, 15 (71.4%) showed a chronic form and 6 (28.6%) showed an acute/subacute form. The incidence of relapse as to clinical form was the same: 15/307 (4.9%) in the CF and 6/93 (6.5%) in the AF (p = 0.60).

Out of the 15 patients in relapse with chronic PCM, 5 of them showed the severe chronic form (SCF), 9 of them showed the moderate chronic form (MCF), and 1 of them showed the mild chronic form (MiCF), while the 6 patients with acute/sub-acute PCM showed the severe acute/subacute form (SAF).

Fourteen out of 21 patients in relapse were treated with TMP-SMX and 7 with ITC. The duration of the treatment with TMP-SMX lasted 33 months, ranging from 28 to 91, and 48 (21–135) with ITC.

These 21 patients showed five diagnosis patterns of relapse: a) positive clinical, serological and mycological – 3; b) positive clinical and mycological – 7; c) positive clinical and serological – 4; d) only positive serological – 2; e) only positive clinical – 5. Regarding as to four of these patterns, in addition to clinical signs, a positive mycological and/or serological test was noted. The five patients who presented only clinical signs were diagnosed as relapse patients based on the exclusion of the other possible etiologies and progress to cure after resumption the treatment with TMP-SMX. In contrast, 2 patients only had a serological relapse, which progressed to a negative response after resuming of the antifungal treatment. The case concerning 9 (47.4%) patients with clinical and/or mycological relapse should be highlighted because the DID tested negative.

Relapses occurred from 46 to 296 months (96 on the average) after treatment introduction and from 4 to 267 months (60 on the average) after treatment discontinuation. The time of relapse did not show any differences compared to the clinical forms: (a) due to treatment introduction: AF = 77.5 months (38–296); CF = 112.0 (46–234); p>0.05; (b) due to treatment discontinuation: AF = 38.5 months (4–267); CF = 75.0 (14–168); p>0.05.

### Specific antibodies serum levels analysis

#### Evaluation of the control group

**Figure 1:**
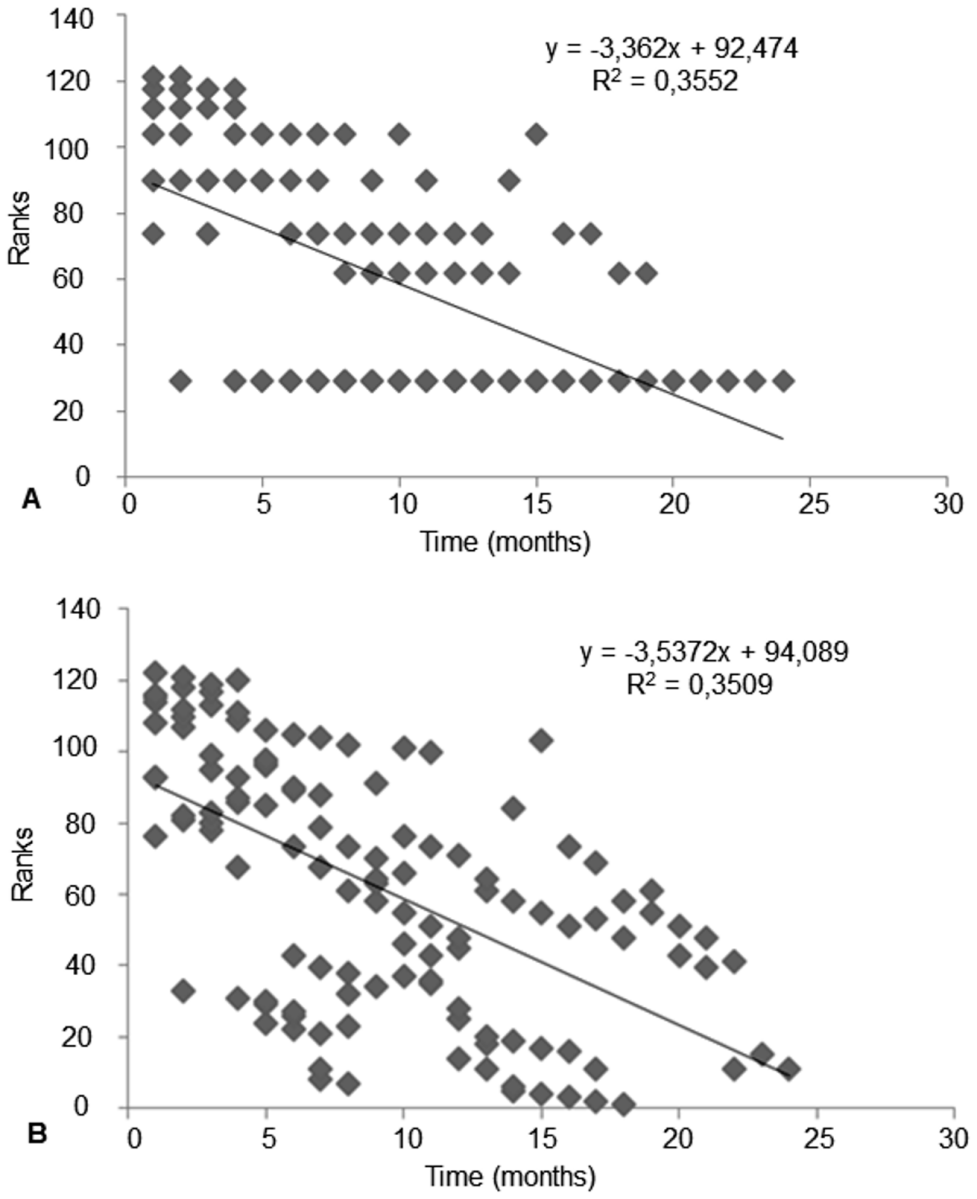
Regression line representing the serum levels variation of anti-*Paracoccidioides brasiliensis* antibodies as a function of antifungal treatment time, by using 122 serum samples from 8 patients who did not relapse with double agar gel immunodiffusion test (DID) positive to admission, before treatment. A – Line standing for double agar gel immunodiffusion test (DID) (p<0.0001) evolution. B – Line standing for enzyme-linked immunosorbent assay (ELISA) (p<0.0001) evolution. Compared under F test, such regressions did not show any difference (p = 0.96).

The serological progress of DID and ELISA in patients who did not relapse showed similar lines reaching negative tests (Figure 1).

The variation of specific antibodies in the serum was evaluated, as ascertained by a reaction to DID and an ELISA test in patients who experienced a relapse, as shown in Table 1. Before treatment, four patients who tested negative for DID also did not show a positive response to the ELISA test. Furthermore, only two inconsistencies in the qualitative results were seen, with one positive response per method. Therefore, the sensitivity of both methods were the same, i.e., equal to 76.1% (p = 0.25).

**Table 1:** Results of the specific antibodies serum levels determined by the enzyme-linked immunosorbent assay (ELISA) and by the double agar gel immunodiffusion test (DID) in 21 patients with relapse of paracoccidioidomycosis.

Patient number	Pretreatment		Time of relapse	
	DID (1:)	ELISA (OD)	DID (1:)	ELISA (OD)
**1**	16	1.441	8	1.345
**2**	128	1.670	4	1.211
**3**	64	1.592	2	0.976
**4**	NR	0.198	—	—
**5**	NR	1.225	2	0.929
**6**	128	1.549	16	1.434
**7**	8	1.325	2	1.006
**8**	256	1.644	NR	0.951
**9**	32	1.356	2	0.853
**10**	2	1.061	NR	0.698
**11**	Undiluted	0.365	NR	0.249
**12**	4	1.224	NR	0.878
**13**	NR	0.606	NR	0.198
**14**	2	0.941	2	0.980
**15**	4	1.216	NR	0.450
**16**	16	1.424	NR	0.790
**17**	NR	0.234	NR	0.163
**18**	256	1.660	NR	0.450
**19**	NR	0.361	NR	0.358
**20**	16	1.403	NR	1.657
**21**	16	1.416	2	0.909

Comparison of the serum levels observed before treatment and at the time of relapse.

— serum not available; NR: nonreactive; cut off/ELISA = 0.710; cut off/DID = NR.

Evaluation of pretreatment/sensitivity: DID = 76.1% ELISA = 76.1% p = 0.25.

Comparison of sensitivity between the pretreatment and time to relapse:

DID: pretreatment = 80% relapse = 45% p = 0.017.

ELISA: pretreatment = 80% relapse = 65% p = 0.125.

Comparing serological test sensitivity at admission, before the introduction of the first treatment, and at the moment of PCM relapse, delivered different results regarding DID and the ELISA test. DID presented a higher sensitivity during pre-treatment (80%) than at the moment of relapse (45%; p = 0.017), while the ELISA test showed an 80% sensitivity in pre-treatment and a 65% sensitivity at relapse, which are not different from each other (p = 0.125). It is important to note that the antibody levels in the serum were, in general, higher at the moment of admission than at the moment of relapse, which is indicated by both DID and ELISA.

Qualitative results of serological methods (DID, ELISA and IB) run in 21 patients with PCM relapse are shown in Table 2. Comparing the serological methods for identifying PCM patients in relapse showed low rates of sensitivity, varying from 12.5% (immunoblotting) to gp70 identification to 65% and 68.8% in the ELISA test (Table 3). The IBgp70 test showed a lower sensitivity than any other test. The ELISA test showed a strong tendency to be more sensitive than DID (p = 0.06) and is as sensitive as IBgp43 (p = 0.11) in diagnosing PCM relapse. Moreover, moderate agreement was noted between DID and ELISA, as illustrated in Table 3.

**Table 2:** Qualitative results of serological tests performed in 20 paracoccidioidomycosis-patients with relapse.

Patients	DID	ELISA	IBgp43	IBgp70
**1**	+	+	NR	NR
**2**	+	+	+	+
**3**	+	+	NR	NR
**4**	—	—	—	—
**5**	+	+	+	NR
**6**	+	+	+	+
**7**	+	+	+	NR
**8**	NR	+	NR	NR
**9**	+	+	NR	NR
**10**	NR	NR	NR	NR
**11**	NR	NR	NR	NR
**12**	NR	+	NR	NR
**13**	NR	NR	NR	NR
**14**	+	+	NR	NR
**15**	NR	NR	—	—
**16**	NR	+	—	—
**17**	NR	NR	+	NR
**18**	NR	NR	+	NR
**19**	NR	NR	—	—
**20**	NR	+	+	NR
**21**	+	+	—	—

Comparison among the double agar gel immunodiffusion test (DID), the enzyme-linked immunosorbent assay (ELISA), and the *immunoblotting* (IB) for identification of the glycoproteins of 43 kDa (IBgp43) and 70 kDa (IBgp70).

— Patients without serum to conduct these tests; NR - nonreactive +: reactive.

**Table 3:** Level of agreement between methods, evaluated 2×2, for serological diagnosis of relapse in paracoccidioidomycosis-patients.

Contrasts		Patients (number)				Sensitivity (%)		Binomial test		kappa coefficient	
A vs B	Total	A+B+	A−B−	A+B−	A−B+	A	B	Level of significance	Value	Confidence interval 95%	Agreement
**DID ** ***vs*** ** ELISA**	20	9	7	0	4	45.0	65.0	p = 0.06	0.6117	[0.2980;0.9253]	Moderate
**DID ** ***vs*** ** IBgp70**	16	4	5	4	3	50.0	37.5	p = 0.23	0.1250	[0.0000;0.6073]	Mild
**DID ** ***vs*** ** IBgp43**	16	2	8	6	0	50.0	12.5	p = 0.016	0.2500	[0.0000;0.5638]	Mild
**IBgp43 ** ***vs*** ** IBgp70**	16	2	9	5	0	43.8	12.5	p = 0.03	0.3103	[0.0000;0.6733]	Mild
**IBgp43 ** ***vs*** ** ELISA**	16	5	3	6	2	43.8	68.8	p = 0.11	0.0448	[0.0000;0.4727]	Mild
**IBgp70 ** ***vs*** ** ELISA**	16	2	5	9	0	12.5	68.8	p = 0.002	0.0122	[0.0000;0.3035]	Mild

Evaluation of the double agar gel immunodiffusion test (DID), the enzyme-linked immunosorbent assay (ELISA) and *immunoblotting* (IB) for identification of the glycoproteins of 43 kDa and 70 kDa. Binomial test and kappa coefficient.

## Discussion

Thus, relapse is defined in this paper as the reappearance of symptomatology suggesting PCM (the etiology of which has been confirmed by mycological and/or serological tests by DID), specific antibodies serum level regression to negative (non-reactive), and persistence of such conditions for at least one year after antifungal therapy discontinuation in patients with prior etiological diagnoses of and successful treatment for PCM, which included clinical cure and the normalization of erythrocyte sedimentation rate. This approach is common in clinical practice, and this paper is aimed at addressing the problem using other methods of serological diagnosis, rather than trying to identifying the source of new infectious processes; otherwise, the research design would have to have been completely different.

The prevalence of relapse out of the 400 patients was equal to 5.2%, i.e., lower than the 13.8% that was observed by Marques [4], who followed up with 58 patients. The prevalence of relapse did not vary according to clinical form, a finding that confirms those of Marques [4]. The diagnosis of relapse was late, i.e., within an average of 60 months after antifungal therapy discontinuation. Marques [4] findings revealed that 50.0% of the reactivations could be observed 36 months after antifungal therapy discontinuation, while our findings presented that 66.6% of the reactivations could be observed after the same period.

Few authors address the subject of PCM relapse, an event that is not rare which can bring serious repercussions to patients, since preserved organs may be impaired and the injured can worsen. In addition, a low prevalence of serological relapse can lead to complications in future diagnoses because it can suggest another manifesting disease, which may lead the MD to opt for unjustified treatments, thus delaying the diagnosis and treatment of the PCM relapse.

This study appraised different serological methods for PCM relapse diagnoses, from the DID reaction, which has been the method of choice due to its specificity, positive predictive value, repeatability and simplicity [6], [17], to immunoblotting with gp43 identification, which to the best of our knowledge and belief, is routinely available only at the Adolfo Lutz Institute (São Paulo). A comparison of both moments, the initial pretreatment and relapse, disclosed that the ELISA test had a higher sensitivity than the DID reaction, which provided several patients with a diagnosis that was not discovered by the gel precipitation test. These findings evince that the ELISA test should be the method of choice for diagnosing PCM relapse. The sensitivity of EIA is still only 65% so a negative result does not rule out relapse infection.

Several mechanisms might explain negative serological results in some relapse patient. One is that the fungus causing the relapse may not be *P. brasiliensis*, a second species could induce the production of DID-undetectable antibodies when using the same antigen as that in the initial reaction. Another explanation would be the modification of the antigenic composition of quiescent fungi, which would initiate the production of modified antibodies that are undetectable by methods designed on the basis of the typical antigens of *P. brasiliensis*.

Two other findings suggest a third hypothesis to explain the absence of antibody detection patients in relapse. The first is the polymorphism of the gene encoding the *P. brasiliensis* gp43 immunodominant antigen [18]. The second is the finding of two different isolates of *P. brasiliensis* in different organs of the same armadillo [19], a finding also observed in a patient with two genetically distinct isolates of *P. brasiliensis*. Clinical isolates were obtained from injuries on different anatomic sites and characterized by means of the RAPD technique. The genotypic evaluation showed more than 28% variability between these fungal isolates, therefore suggesting that different genotypes of *P. brasiliensis* may infect the same patient and induce active disease [20]. Thus, it is possible that some patients have been infected by more than one of *P. brasiliensis* isolate, with a positive serology to one of them; the other isolate, responsible for the relapse, could not be serologically recognized [21].

Other studies confirm these hypotheses. The cryptic species of *Paracoccidioides brasiliensis*, S1, PS2, PS3 and *Paracoccidioides lutzii*, recently identified [22]–[24], have implications on PCM immunodiagnosis [25]–[26], and presented a regional distribution. Such results suggest that there are differences in the fungus antigenic composition of *P. brasiliensis*. Arantes et al. [27] collected aerosol samples for the environmental detection of *Paracoccidioides ssp.*, by placing a cyclonic air sampler at the entrance of the armadillo burrow in Botucatu (SP). Most ITS sequences showed a high similarity with homologous sequences of *P. lutzii* in the GenBank database, suggesting that this species – *Paracoccidioides lutzii* – may not be exclusive in Midwest Brazil.

Studies involving other microorganisms also confirm such hypotheses. Ghannoum et al. [28] analyzed clinical isolates of *Cryptococcus neoformans* obtained from five patients with recurrent *cryptococcal meningitis* and demonstrated the different composition of sterols between relapse and pre-treatment isolates, which indicates that the sterols had been modified by therapy or that patients were infected with new isolates with different sterol compositions.

Soll et al. [29] monitored *Candida albicans* strains isolated from different body sites of a single patient in three events of recurrent vulvovaginal candidiasis. The strains were evaluated by using Southern blot hybridization. They observed three different strains of *C. albicans* colonizing five sites, at the time of the first infection. Although the same strain of *C. albicans* had been responsible for the three vaginal infections, different colony phenotypes manifested with every new infection.

Such findings suggest that the action of antifungal and/or immune response of patients could lead to changes in the antigenic composition of fungi that are responsible for relapse, which would induce the production of antibodies that do not recognize the antigens used in routine serological tests. Hence, diagnostic methods based on proteomics could contribute to the solution of this problem. Evaluations in that direction are already in the course of pilot study in our Service. Another possible method of diagnosis of PCM relapse would be the analysis of circulating [30] and/or urinary [31] antigens, the methods of which have been evaluated successfully in the initial diagnosis of this mycosis and in follow-up with these patients. Nevertheless, such methods are not yet routinely available in clinical laboratories, especially those that are public.

Polymerase Chain Reaction (PCR) with primers specific for *P. brasiliensis* detection in clinical materials and other molecular method could also be excellent options for diagnosing PCM relapse, however, these techniques have not been incorporated yet into the routine of clinical laboratories [32]. In addition, PCR sensitivity is low in serum samples [33], the aim of our study, although higher in other clinical materials [32]–[35].

As a final point, while more sensitive and specific serological methods are assayed, we should continue to give special attention to the mycological diagnosis of PCM relapse. Such methods include the gold standard of diagnosing PCM. Hence, direct mycological [36]–[37], cytopathological [36]–[38], histopathological [36] and cultivation tests for *P. brasiliensis* [36]–[39] must be appropriately and routinely conducted when following up with such patients.
